# A Safe and Easy Introduction of Darbepoetin-Alpha in Patients Receiving Maintenance Hemodialysis and Epoetin Monotherapy: A “Half-and-Half” Combination Therapy^☆^

**DOI:** 10.1016/j.curtheres.2012.12.001

**Published:** 2013-06

**Authors:** Kazumasa Shimamatsu, Hiroko Inamasu

**Affiliations:** Shimamatsu Naika Iin, Shiseikai Medical Corporation, Chikushino City, Fukuoka, Japan

**Keywords:** combination therapy, darbepoetin, epoetin, hemodialysis patients

## Abstract

**Background:**

In hemodialysis (HD) patients requiring anemia management, the 3-fold longer terminal half-life (25.3 hours) of darbepoetin-alpha (DA) results in reduced dose frequency when compared with recombinant human erythropoietin (EPO) -alpha or -beta by intravenous administration (8.5 hours). However, this might become a disadvantage in the face of rapid withdrawal of the drug against hemoglobin (Hb) overshoot and/or cycling.

**Objective:**

A “half-and-half” combination therapy of DA and EPO was used to avoid a possible Hb overshoot due to the full conversion from EPO to DA.

**Methods:**

Thirty-two stable patients receiving HD (13 men, 19 women) and EPO monotherapy were enrolled and prospectively followed for 9 months. The mean (SD) patient age was 63.2 (11.3) years. The HD duration was 10.7 (8.2) years. The DA doses (in micrograms) of 1/200 of halves of previous weekly EPO doses (in international units) were given intravenously on the second HD day of a week. The remaining half doses of previous weekly EPO doses were dividedly administered intravenously on the first and the third HD days of the week. The target Hb was 11 g/dL.

**Results:**

The “half-and-half” combination with DA and EPO resulted in no episodes of Hb overshoot. The Hb values did not exceed 13 g/dL throughout the follow-up period. The mean (SD) dose of 3984 (2175) IU/wk EPO was converted to a combination of 1688 (894) IU/wk EPO and 13.4 (7.9) μg/wk DA at baseline. Thereafter, the mean (SD) doses became 304 (656) IU/wk EPO and 16.0 (8.4) μg/wk DA at 3 months, and 532 (912) IU/wk and 15.8 (9.0) μg/wk, respectively, at 9 months. The total combination doses of DA/EPO (as EPO equivalents) were significantly reduced to 80% to 84% of the original EPO doses after 2 months of introduction of the DA/EPO combination.

**Conclusions:**

A “half-and-half” combination therapy may be a safe and easy method to merge DA into EPO monotherapy without Hb overshoot or dramatic cycling.

## Introduction

Since the late 1980s, recombinant human erythropoietin (also known as epoetin [EPO]) has played a critical role in the management of renal anemia in patients receiving maintenance dialysis, resulting in prolonged survival rates and improved quality of life in addition to decreased need for blood transfusions.^1–3^ A decade later, darbepoetin-alpha (DA), which has 5 N-linked carbohydrate chains and 2 added N-linked carbohydrate sites in the primary sequence (3 N-linked chains) of EPO, was developed for clinical use with a 3-fold longer mean terminal half-life of 25.3 hours for intravenous DA compared with 8.5 hours for intravenous EPO.^4–6^ In fact, DA administered once weekly is as effective and well tolerated as EPO administered 3 times weekly for the treatment of anemia in patients not receiving dialysis or patients with chronic kidney disease who are receiving dialysis.^7–10^ Its longer terminal half-life results in reduced dose frequency relative to EPO; however, this might simultaneously become a disadvantage in the face of rapid withdrawal of the drug against hemoglobin (Hb) overshoot and/or cycling.^11,12^

When DA, which became commercially available in July 2007 in Japan, was introduced to our clinic during December 2007, halves of weekly EPO doses were converted to DA (“half-and-half” combination therapy) to avoid a possible overshoot in Hb rise with its full conversion from EPO to DA. This is the first report of combination therapy with DA and EPO in patients receiving maintenance hemodialysis (HD).

## Patients and Methods

**Figure 1:**
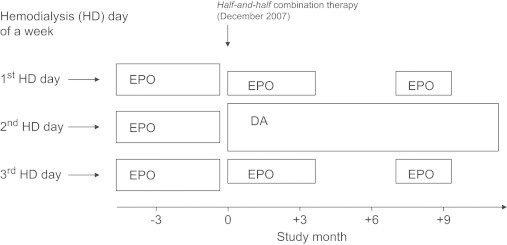
Schematic image of “half-and-half” combination therapy with darbepoetin (DA) and epoetin (EPO). Preparations (see the text) of DA close to the doses (in micrograms) of 1/200 of halves of weekly EPO doses (in international units) were chosen from the lineups and given intravenously on the second hemodialysis (HD) day of the week. The remaining half doses of EPO were divided and given intravenously on the first and the third HD days of the week.

Although both EPO and DA had been used in clinical practice, informed consent for this study was obtained by mouth from all patients enrolled. Thirty-two stable outpatients receiving HD and EPO monotherapy who had no episodes of bleeding complications and/or admissions for an antecedent 3 months were selected with informed consent by word of mouth during December 2007 for DA/EPO combination therapy and prospectively followed until September 2008. All patients selected were already stabilized with intravenous EPO monotherapy for a mean (SD) extended period of 10.7 (8.2) years before September 2007. HD consisted of 5-hour treatments performed 3 times per week. When DA was introduced to our clinic during December 2007, halves of weekly EPO doses were converted to DA by intravenous injection. The drug preparations available to our clinic were as follows: 10, 20, and 40 μg DA (Nesp; Kyowa Hakko Kirin Co Ltd, Tokyo, Japan) and 750, and 1500 IU EPO (Epogin; Chugai Pharmaceutical Co Ltd, Tokyo, Japan). Preparations of DA close to the doses (in micrograms) of 1/200 ^5,7–9^ of halves of weekly EPO doses (in international units) were chosen from among the lineups and given intravenously on the second HD day of each week. The remaining half doses of EPO were divided and administered intravenously on the first and the third HD days of the week (Figure 1). The target Hb level was 11 g/dL. After introduction of DA combined with EPO, EPO was eliminated when the Hb levels rose to >12 g/dL. In cases of unchanged Hb levels DA doses were increased. When the Hb levels decreased to 10 g/dL after elimination of EPO, EPO was added again to augment the DA treatment (Figure 1).

Intravenous iron supplementation (ferric oxide, saccharated) (Fesin; Nichi-Iko Co. Ltd, Toyama, Japan) was also administered. When serum ferritin levels fell to <100 ng/mL, 40 mg iron was administered intravenously once a week. Once serum ferritin levels reached >100 ng/mL, 40 mg iron was given intravenously twice a month and adjusted quarterly (during March, June, September, and December) according to serum ferritin measurements.^13,14^ When the quarterly serum ferritin values exceeded 300 ng/mL the frequency of intravenous iron administration was decreased and when they reached 500 ng/mL, iron supplementation was discontinued. A complete blood count was performed twice monthly. The serum ferritin concentration and the total iron-binding capacity were determined quarterly, and the serum iron concentration was measured monthly along with the other routine general blood tests. The transferrin saturation (TSAT) was calculated using the following formula: (serum iron concentration/total iron-binding capacity)×100%. All data are presented as mean (SD). Student *t* test (paired and unpaired) was applied for statistical analysis. Differences were considered to be statistically significant at *P* values <0.05. Data from December 2007 were used as baseline values.

## Results

The mean (SD) age of patients (13 men, 19 women) was 63.2 (11.3) years. The mean (SD) HD duration was 10.7 (8.2) years. The underlying renal diseases were chronic glomerulonephritis in 17 patients, diabetic nephropathy in 7 patients, hypertensive nephrosclerosis in 3 patients, chronic pyelonephritis in 2 patients, polycystic kidney disease in 2 patients, and Alport syndrome in 1 patient.

**Figure 2:**
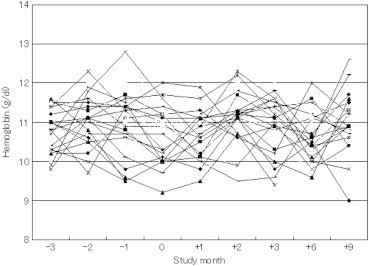
Serial changes in hemoglobin (Hb) values in 32 maintenance hemodialysis (HD) patients before and after the initiation of “half-and-half” combination therapy with darbepoetin (DA) and epoetin (EPO). During the first 3 months of the transition from EPO monotherapy to “half-and-half” combination therapy of DA/EPO, only a few data exceeded Hb level of 12 g/dL. All Hb levels were <13 g/dL.

**Figure 3:**
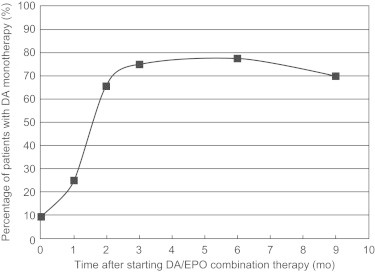
Percentage of patients with darbepoetin (DA) monotherapy after initiation of “half-and-half” combination therapy with darbepoetin/epoetin (DA/EPO).

As shown in the Table, before and after converting the EPO monotherapy to the “half-and-half” combination therapy of DA and EPO, mean Hb levels remained unchanged at around 11 g/dL of the Hb target during the study period. However, the Hb levels in November 2007 and in February, March, and September 2008 were significantly higher than that observed in December 2007 (baseline). During the combination therapy there were no episodes of Hb overshooting (>3 g/dL^11,12^). As shown in Figure 2, the actual Hb values did not exceed 13 g/dL throughout the follow-up period. The mean (SD) dose of EPO, which was 3984 (2175) IU/wk in November 2007, was converted to the combination therapy with 1688 (894) IU/wk EPO and 13.4 (7.9) μg/wk DA in December 2007. Thereafter, the mean doses of the combination became 304 (656) IU/wk EPO and 16.0 (8.4) μg/wk DA in March 2008, and 532 (912) IU/wk and 15.8 (9.0) μg/wk (n = 31), respectively, in September 2008. The total doses of DA/EPO as EPO equivalents were significantly reduced to 80% to 84% of baseline EPO dose after February 2008 (see Table). Three months after the combination therapy, approximately 70% of patients were receiving DA alone and 30% of patients were receiving combination therapy (Figure 3). Mean (SD) serum ferritin concentration and TSAT did not significantly change throughout the study period: 237 (82) ng/mL serum ferritin concentration and 31.0% (11.3%) of TSAT in September 2007, 253 (83) ng/dL serum ferritin concentration and 30.5% (10.5%) of TSAT in December 2007 (at the start of the “half-and-half” combination), and 239 (109) ng/mL serum ferritin concentration and 33.1% (12.5%)of TSAT (n = 31) in September 2008.

## Discussion

Despite the irreplaceable effects of EPO or DA on anemia management in patients with CKD,^1–3,7–9^ recent large-scale randomized controlled trials^15–18^ strongly suggested harmful effects of higher doses of EPO or DA per se as well as higher Hb levels close to normal (>13 g/dL). Our approach using “half-and-half” combination of DA and EPO may be a safe and easy way to introduce DA without overshooting Hb levels beyond the ranges recommended.^19,20^ In the study for comparing DA with EPO on anemia management in patients with chronic renal failure not yet on dialysis reported by the European/Australian Novel Erythropoiesis Stimulating Protein Study Group,^7^ Hb overshooting >14 g/dL developed in 24% of patients treated with DA and in 35% of patients treated with EPO. The combination of DA/EPO therapy proposed in our study might become a solution of potential concern for Hb overshooting on EPO or DA monotherapy.

Although the conversion ratio from EPO to DA was originally recommended to be 1:200 (1 μg DA = 200 IU EPO),^5,7–9^ revised ratios of 1:250 to 350 have recently been reported.^6,21–23^ In our study, in which the original conversion ratio of 1:200 was adopted, the virtual conversion ratio became 1:400 because the half dose of EPO was converted to DA. The approach proposed here for initiation of DA may be safe and easy for those who are not familiar with the drug and prefer to avoid the drastic conversion from EPO to DA. In our study, although the total doses (as the equivalent of EPO) of DA with EPO were reduced to between 80% and 84% of the baseline EPO dose during the period of EPO monotherapy, the final mean (SD) doses of the DA/EPO combination therapy were 15.8 (9.0) μg/wk DA and 532 (912) IU/wk EPO versus the baseline weekly doses of 3984 (2175) IU/wk having resulted in the consequent conversion ratio of 1:220 (see the Table). Interestingly, Bock et al^22^ reported that although the equimolar 1:200 conversion ratio was appropriate for lower EPO doses (<5000 IU/wk) DA dose for patients converting from ≥5000 IU/wk EPO was more likely to follow a 1:250 to 1:350 conversion rule. Our DA/EPO combination method may be, in this context, more useful for patients receiving EPO monotherapy ≥5000 IU/wk.

Our study also showed that the small lineups of 10, 20, and 40 μg DA and 750, and 1500 IU EPO were enough to carry out the “half-and-half” combination therapy. Further, by designating the days of a week to apply the preparations (the second HD day of a week for DA and the first and third HD days of a week for EPO), no staff members made injection mistakes during the study period (Figure 1).

In addition, iron indices of serum ferritin concentration and TSAT were not changed after introduction of DA in our study, consistent with the results from other studies.^9,23^ A longer period of observation will, however, be needed to verify whether or not the iron requirement is changed by introduction of DA.

## Conclusions

A “half-and-half” combination therapy may be a safe and easy method to merge DA into EPO monotherapy without a concern about an overshoot in Hb. Further, its long-term effect on anemia management in HD patients is worth testing.

## Conflicts of Interest

The authors have indicated that they have no conflicts of interest regarding the content of this article.

## Figures and Tables

**Table:** The serial changes of epoetin and darbepoetin doses (including total doses as epoetin equivalents), hemoglobin value, serum ferritin concentration, and transferrin saturation (TSAT) in 32 maintenance hemodialysis patients before and after the introduction of darbepoetin in December 2007.*

	2007				2008				
	September	October	November	December	January	February	March	June	September
	Study Month								
	−3	−2	−1	0 (Baseline)	+1	+2	+3	+6	+9
n	32	32	32	32	32	32	32	31†	31†
Epoetin beta (IU/wk)	4148 (1797)	4148 (2007)	3984 (2175)	1688 (894)	1172 (951)	445 (734)	304 (656)	339 (720)	532 (912)§
Darbepoetin alpha (μg/wk)	0	0	0	13.4 (7.9)	16.7 (9.1)	15.8 (8.5)	16.0 (8.4)	16.6 (8.1)	15.8 (9.0)
Total doses as epoetin equivalent (IU/wk)‡	4148 (1797)	4148 (2007)	3984 (2175)	4375 (2136)	4516 (2300)	3602 (1831)§	3510 (1753)§	3661 (2030)∥	3694 (2400)§
Hemoglobin (g/dL)	10.8 (0.6)	11.0 (0.7)	11.0 (0.8)§	10.7 (0.7)	10.8 (0.6)	11.2 (0.7)§	11.1 (0.7)§	10.8 (0.7)	11.0 (0.7)§
Serum ferritin (ng/mL)	237 (82)	Not done	Not done	253 (83)	Not done	Not done	232 (96)	227 (88)	239 (109)
TSAT (%)	31.0 (113)	Not done	Not done	30.5 (10.5)	Not done	Not done	31.6 (16.6)	30.8 (13.0)	31 (12.5)

⁎ Data are expressed as mean (SD).

† The data deficit (n = 31) in June and September 2008 resulted from an admission of a male patient (aged 77 years) who had an accidental contusion of the head in the bathroom accompanied by intracranial bleeding.

‡ Total doses as epoetin equivalents are calculated using the formula [epoetin dose + (200 × darbepoetin dose)].

§ *P* <0.05.

∥ *P* <0.01 (against the baseline data of December 2007).
